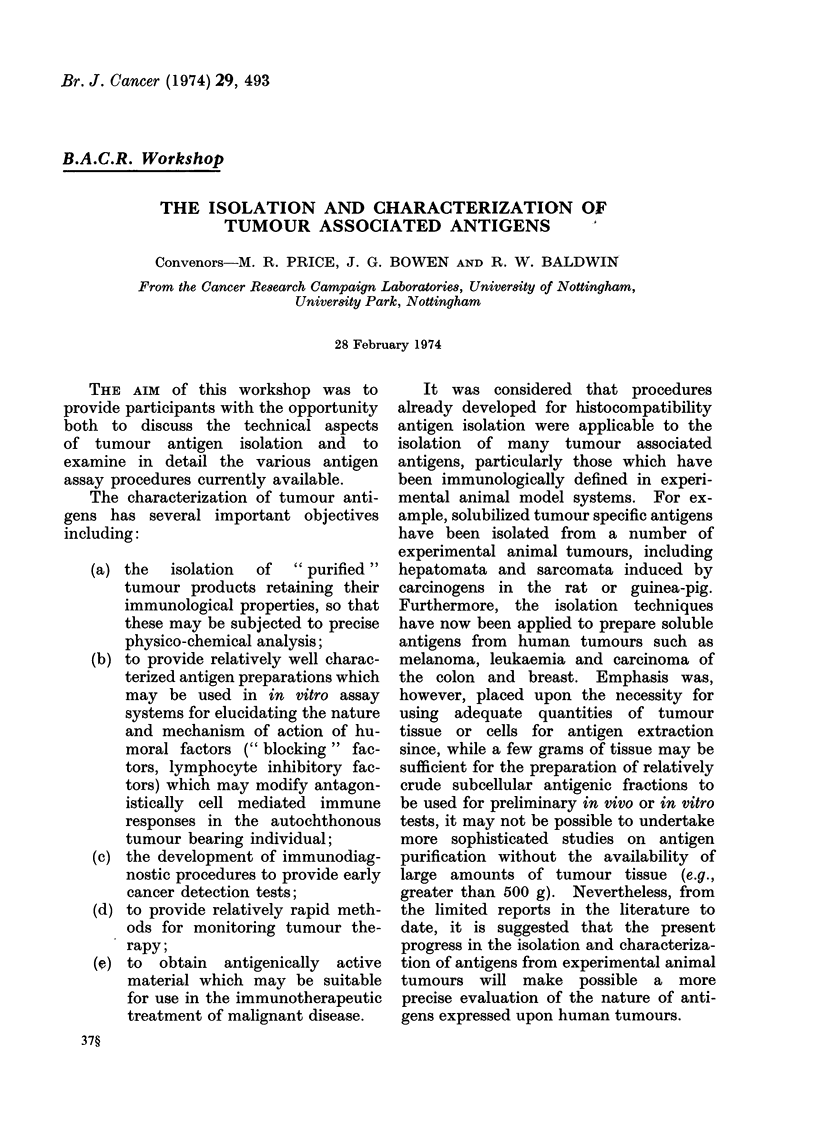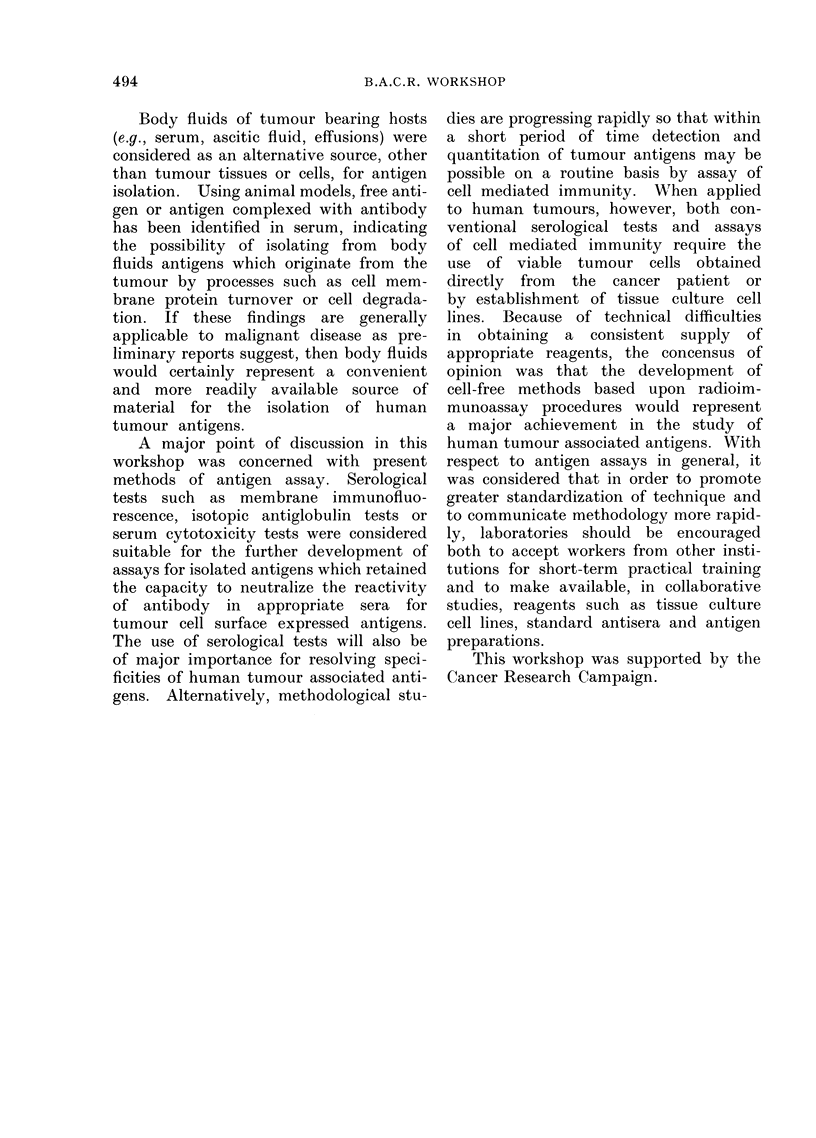# The isolation and characterization of tumour associated antigens.

**DOI:** 10.1038/bjc.1974.105

**Published:** 1974-06

**Authors:** M. R. Price, J. G. Bowen, R. W. Baldwin


Br. J. Cancer (1974) 29, 493

*B.A.C.R. Workshop*

### THE ISOLATION AND CHARACTERIZATION OF TUMOUR ASSOCIATED ANTIGENS

Convenors—M. R. PRICE, J. G. BOWEN AND R. W. BALDWIN

*From the Cancer Research Campaign Laboratories, University of Nottingham, University Park, Nottingham*

28 February 1974

THE AIM of this workshop was to provide participants with the opportunity both to discuss the technical aspects of tumour antigen isolation and to examine in detail the various antigen assay procedures currently available.

The characterization of tumour antigens has several important objectives including:

(a) the isolation of "purified" tumour products retaining their immunological properties, so that these may be subjected to precise physico-chemical analysis;

(b) to provide relatively well characterized antigen preparations which may be used in *in vitro* assay systems for elucidating the nature and mechanism of action of humoral factors ("blocking" factors, lymphocyte inhibitory factors) which may modify antagonistically cell mediated immune responses in the autochthonous tumour bearing individual;

(c) the development of immunodiagnostic procedures to provide early cancer detection tests;

(d) to provide relatively rapid methods for monitoring tumour therapy;

(e) to obtain antigenically active material which may be suitable for use in the immunotherapeutic treatment of malignant disease.

It was considered that procedures already developed for histocompatibility antigen isolation were applicable to the isolation of many tumour associated antigens, particularly those which have been immunologically defined in experimental animal model systems. For example, solubilized tumour specific antigens have been isolated from a number of experimental animal tumours, including hepatomata and sarcomata induced by carcinogens in the rat or guinea-pig. Furthermore, the isolation techniques have now been applied to prepare soluble antigens from human tumours such as melanoma, leukaemia and carcinoma of the colon and breast. Emphasis was, however, placed upon the necessity for using adequate quantities of tumour tissue or cells for antigen extraction since, while a few grams of tissue may be sufficient for the preparation of relatively crude subcellular antigenic fractions to be used for preliminary *in vivo* or *in vitro* tests, it may not be possible to undertake more sophisticated studies on antigen purification without the availability of large amounts of tumour tissue (*e.g.*, greater than 500 g). Nevertheless, from the limited reports in the literature to date, it is suggested that the present progress in the isolation and characterization of antigens from experimental animal tumours will make possible a more precise evaluation of the nature of antigens expressed upon human tumours.

Body fluids of tumour bearing hosts (*e.g.*, serum, ascitic fluid, effusions) were considered as an alternative source, other than tumour tissues or cells, for antigen isolation. Using animal models, free antigen or antigen complexed with antibody has been identified in serum, indicating the possibility of isolating from body fluids antigens which originate from the tumour by processes such as cell membrane protein turnover or cell degradation. If these findings are generally applicable to malignant disease as preliminary reports suggest, then body fluids would certainly represent a convenient and more readily available source of material for the isolation of human tumour antigens.

A major point of discussion in this workshop was concerned with present methods of antigen assay. Serological tests such as membrane immunofluorescence, isotopic antiglobulin tests or serum cytotoxicity tests were considered suitable for the further development of assays for isolated antigens which retained the capacity to neutralize the reactivity of antibody in appropriate sera for tumour cell surface expressed antigens. The use of serological tests will also be of major importance for resolving specificities of human tumour associated antigens. Alternatively, methodological studies are progressing rapidly so that within a short period of time detection and quantitation of tumour antigens may be possible on a routine basis by assay of cell mediated immunity. When applied to human tumours, however, both conventional serological tests and assays of cell mediated immunity require the use of viable tumour cells obtained directly from the cancer patient or by establishment of tissue culture cell lines. Because of technical difficulties in obtaining a consistent supply of appropriate reagents, the concensus of opinion was that the development of cell-free methods based upon radioimmunoassay procedures would represent a major achievement in the study of human tumour associated antigens. With respect to antigen assays in general, it was considered that in order to promote greater standardization of technique and to communicate methodology more rapidly, laboratories should be encouraged both to accept workers from other institutions for short-term practical training and to make available, in collaborative studies, reagents such as tissue culture cell lines, standard antisera and antigen preparations.

This workshop was supported by the Cancer Research Campaign.